# The box task - a method for assessing in-vehicle system demand

**DOI:** 10.1016/j.mex.2021.101261

**Published:** 2021-02-03

**Authors:** Daniel Trommler, Tina Morgenstern, Elisabeth M. Wögerbauer, Frederik Naujoks, Josef F. Krems, Andreas Keinath

**Affiliations:** a Chemnitz University of Technology, Chemnitz, Germany; b Stuttgart Media University, Stuttgart, Germany; c BMW Group, Munich, Germany

**Keywords:** Box Task, Evaluation methods, Secondary task demand, In-vehicle information systems

## Abstract

The use of advanced in-vehicle information systems (IVIS) and other complex devices such as smartphones while driving can lead to driver distraction, which, in turn, increases safety-critical event risk. Therefore, using methods for measuring driver distraction caused by IVIS is crucial when developing new in-vehicle systems. In this paper, we present the setup and implementation of the Box Task combined with a Detection Response Task (BT+DRT) as a tool to assess visual-manual and cognitive distraction effects. The BT+DRT represents a low-cost and easy-to-use method which can be easily implemented by researchers in laboratory settings and which was validated in previous research. Moreover, at the end of this paper we describe the experimental procedure, the data analysis and discuss potential modifications of the method.

• The setup and implementation of the Box Task combined with a Detection Response Task (BT+DRT) is described.
• The method allows for measuring visual-manual and cognitive distraction of drivers.
• The BT+DRT is a cost-effective and easy-to-use method that can be implemented in laboratory settings or driving simulators.

## Graphical abstract

**Figure:**
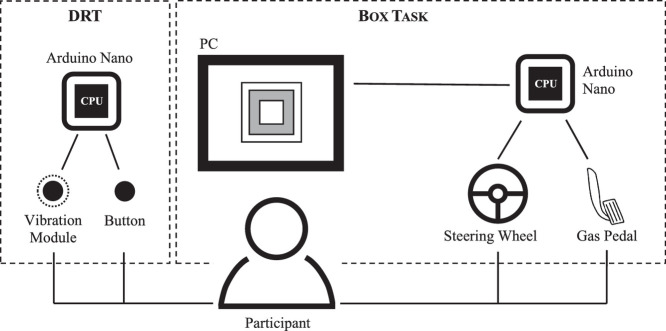

Specifications Table

**Table:** 

Subject Area	Psychology
More specific subject area	Human Factors
Method name	Box Task + Detection Response Task
Name and reference of original method	Hsieh, L., & Seaman, S. (n.d.). Evaluation of the Two-Dimensional Secondary Task Demand Assessment Method. Unpublished report, Department of Communication Sciences and Disorders, Wayne State University.
Resource availability	https://mytuc.org/mvml

## Method overview

### Rationale

Using information and communication technologies while driving has increased strongly in recent years. In a survey study by Kubitzki and Fastenmeier [6], about 47% of the surveyed German, Austrian and Swiss drivers reported that they use their cell phone while driving and about 75% of the drivers indicated to be distracted by technologies integrated into the vehicle such as infotainment systems. However, it is well known that especially visual-manual secondary tasks, such as manipulating a handheld cell phone, are associated with an increased safety critical event risk due to the long off-road glances [10]. Hence, it is the responsibility of car manufacturers to ensure that modern information and communication technologies integrated into the vehicles meet test criteria that are associated with limited distraction potential. In addition, more and more drivers use portable electronic devices (e.g., cell phones) while driving (see e.g., [6]), which can have adverse effects on driving performance. A valid, reliable and cost-effective method to assess secondary task demand is therefore crucial for the development of new (in-vehicle) systems.

In the beginning of the 21^st^ century, there was a lot of research regarding the question how to accurately measure secondary task demand while driving. Some methods have been developed that focused either mainly on visual (e.g., occlusion technique, see [5]) or on cognitive distraction effects (e.g., detection response task (DRT), see [4]). There are only a few methods that try to cover both aspects combined (e.g., lane change task (LCT), see [3]).

Regarding the impact of cognitive distraction on driving performance, previous research is rather inconsistent. Engström et al. [1] try to explain these controversial findings using the cognitive control hypothesis. Thus, cognitive load has different effects on driving performance depending on the automation of drivers’ task performance. Automated tasks are based on practice and experience and require less attention. Hence, such tasks are effortless to perform (e.g., steering to correct course errors while staying in lane). The performance of these tasks is therefore largely unaffected by cognitive load or, in some cases, even improved. In contrast, novel, non-routine tasks require more attention and cognitive control. As a result, performance is more affected by cognitive load. To avoid that driving experience affects the assessment of cognitive distraction effects, artificial laboratory tasks (instead of real driving performance parameters) should be used. These tasks are usually unfamiliar to participants, or at least less practiced, and should therefore capture effects of cognitive load more reliably [1].

According to the Dimensional Model of Driver Demand (see [11]), driving task demand can be divided into two components: Physical and cognitive demand. Physical demand is related to lateral and longitudinal driving task performance, whereas cognitive demand refers to the ability to detect events in a timely manner. However, existing methods that combine physical (or visual-manual) as well as cognitive demand, such as the LCT, are not or only in a very limited way able to assess the impact of different distraction effects separately. Such a separation can be useful to develop or optimize infotainment systems in a way to reduce driver distraction as much as possible.

A new method that is based on the Dimensional Model of Driver Demand [11] is the Box Task in combination with the DRT (for more details see [2]). Initial studies have shown that this method is sensitive to visual-manual as well as cognitive distraction effects [7,8]. This paper presents the setup and application of the Box Task with C# and Unity for Microsoft Windows and shows possibilities for evaluating different secondary tasks with the Box Task in combination with a DRT (BT+DRT).

## Method details

**Fig. 1:**
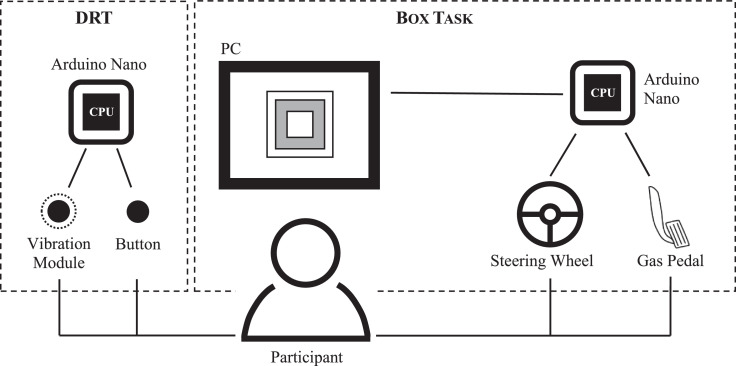
Setup of the Box Task and DRT. In addition to the widely used DRT, participants interact with a steering wheel and gas pedal to perform the Box Task.

The setup of the method follows the differentiation of the Dimensional Model of Driver Demand [11] into physical and cognitive demand. Thus, physical demand is assessed by the BT and cognitive demand by the DRT. The setup is shown in Fig. 1.

The DRT is an ISO-standardized test procedure [4]. Within the present method, a tactile DRT is used. A vibration module placed on participants‘ shoulders gives a tactile signal (vibration) in a random interval of three to five seconds. Participants have to react to this signal by pressing a button at the steering wheel. To get an indication about cognitive demand, hit rate and mean reaction time are calculated (for more details see [4]).

**Fig. 2:**
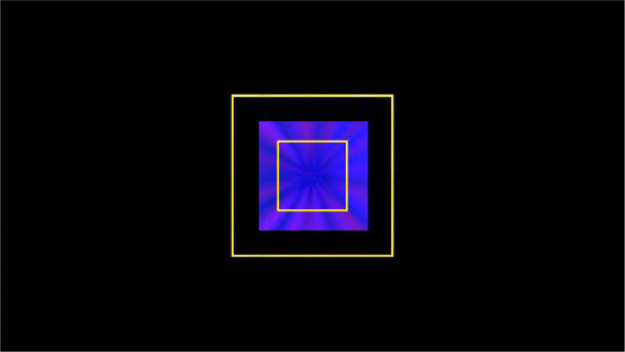
Box Task (BT), example screen. The blue box's position and size change continuously. The two yellow squares represent the guide boxes (i.e., inner and outer boundaries).

The BT simulates a car-following scenario. Within the BT, a displayed box changes its lateral position (~ lane maintenance) and size (~ headway to a lead vehicle) continuously in a sinusoidal pattern. Participants have to adjust to these changes by moving the steering wheel (lateral position of the box) and the gas pedal (size of the box). That means, participants are instructed to keep the box within an inner and outer boundary (see Fig. 2). To get an indication about visual-manual demand, error states are calculated.

### Setup of the BT

Our software is implemented with C# and Unity for Microsoft Windows (window size: 1024 × 768 pixels). The size of the inner boundary is 150 pixels, the size of the outer boundary is 350 pixels. Hence, the ratio of inner to outer boundary is 0.43.

Based on the approach of Hsieh and Seaman (n.d.), size and position of the box are continuously influenced by a time-dependent function ($f_{size}\;and\;f_{position})\;$ and by participants’ input via the angle of the gas pedal ($angle_{pedal}$) and steering wheel ($angle_{wheel}$).

$$size=size+f_{size}\left(time\right)+angle_{pedal}$$

$$position=position+f_{position}\left(time\right)+angle_{wheel}$$

The box changes its size and position continuously in a sinusoidal pattern:

$$f_{size}\left(time\right)=\sin\left(\frac{0.625\times \pi \times time}{60}\right)\times 0.15$$

$$f_{position}\left(time\right)=\sin\left(\frac{0.750\times \pi \times time}{60}\right)\times 0.18$$

time = elapsed time since starting

In the present setting, the sine wave of the box completes 0.625 longitudinal (box size) and 0.750 lateral (box position) cycles per minute. By adjusting the value domain of the sinus functions, $f_{size}\;$ reaches an amplitude of 72 pixels (see maxSizeOffsetPerc = 0.15) and $f_{position}$ an amplitude of 145 pixels (see maxLatOffsetPerc = 0.18).

Participants are instructed to use the steering wheel to adjust the box position. By rotating the steering wheel to the left/right, the box also moves to the left/right. Analogously, participants need to increase/decrease pressure on the gas pedal to increase/decrease the box size.

The gas pedal and the steering wheel are equipped with a sensor (MPU-6050) which combines a 3-axis gyroscope (we use a full scale range of ±500 °/sec) and 3-axis accelerometer (we use a full scale range of ±4g) to measure the deflection of both. Thus, pressure on the gas pedal and movement of the steering wheel can be detected. The values of the accelerometer (ax, ay, az) and gyroscope (gx, gy) are collected by a microcontroller (we use Arduino Nano), which is connected to the sensors. After dividing the raw values by a range-specific factor (depending on the used programming library of the sensor, in our case 8.192 for accelerometer and 65.5 for gyroscope), these values are submitted to the Box Task software via USB interface. The implementation through external sensors allows the use of the BT in different settings.

The angle of the steering wheel and gas pedal is a result of the values of gyroscope (GyrData) and accelerometer (AccData) which are integrated partially through a complementary filter. Using the accelerometer data avoids a drift of the values, which can occur when using the gyroscope over a long term. For every time step, the gyroscope data is added to the current angle. We use a ratio of 98% of gyroscope data and 2% of accelerometer data. The complementary filter has the following equation:

angle=0.98×(angle+GyrData×dt)+0.02×AccData

angle = current angular value
GyrData= Gyroscope data
dt = Delta T (time since last frame)
AccData = Accelerometer data

From this equation, the calculation of the deflection of the gas pedal and rotation of the steering wheel can be derived. The accelerometer data are previously low-pass filtered with atan2 for reliable data.

$$angle_{pedal}=0.98\times \left(angle_{pedal}-\left(gas_{gy}\times dt\right)\right)+0.02\times \left(\frac{atan2\left(gas\_ax,\;gas\_az\right)\times 180}{\pi }\right)$$

$$angle_{wheel}=0.98\times \left(angle_{wheel}+\left(wheel_{gx}\times dt\right)\right)+0.02\times \left(\frac{atan2\left(wheel\_ay,\;wheel\_az\right)\times 180}{\pi }\right)$$

After integrating the sinus value and participants’ input value, the results are converted into pixels to determine the new box position and size. Considering the maximum and minimum size as well as the maximum left and right position, the box is adjusted and displayed accordingly.

### Experimental procedure

To assess the distraction potential of secondary tasks/in-vehicle systems with the BT+DRT, an experimental study has to be conducted. For our studies (see [7,8]), we used a laboratory setting. Hence, the participants sat in front of a computer/monitor with a gas pedal and a steering wheel. They were informed that the Box Task represents the primary task, i.e., they should “drive” as safely as possible while simultaneously engaging in different secondary tasks. The primary and secondary tasks were explained by written instructions. Before each test block, participants could practice the BT+DRT until they felt comfortable. Afterwards, participants had to perform a baseline run (i.e., BT+DRT without secondary task engagement) and the dual-task conditions.

Participants had to use the gas pedal und steering wheel to keep the box within the boundaries. Within each trial, participants had 30 seconds to get familiar with the BT. After 30 seconds, the dual-task conditions started. An exclamation mark and a beep sound were presented as a prompt to start with secondary task engagement. Additionally, participants had to respond to the haptic signal of the DRT. The experimental trials ended after 210 seconds.

This procedure was repeated for all dual-task conditions. For more details, see Morgenstern, Wögerbauer et al. [7] and Morgenstern, Trommler et al. [8].

### Data analysis

**Fig. 3:**
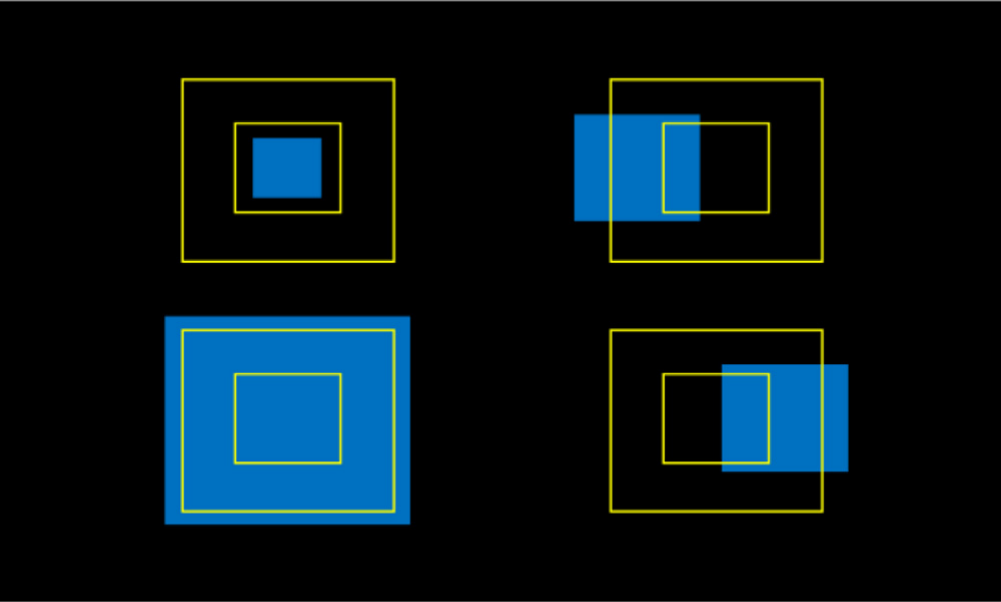
Examples of longitudinal (left) and lateral (right) error states.

Participants’ BT performance can be divided into lateral and longitudinal errors. Longitudinal errors occur when the dynamic box leaves the boundaries, i.e., the box becomes either smaller than the inner boundary or larger than the outer boundary (see Fig. 3, left). A lateral error exists if the size of the dynamic box is within the boundaries, but exceeds the outer boundary either on the left or on the right side (see Fig. 3, right). In addition to the number and/or duration of lateral and longitudinal error states, the variability of the box size and the box position is assessed. For this, the standard deviation from the ideal box size and position is used.

For each trial, a logging file is created. Next to a header (i.e., time of experiment and participants’ ID), the following data are stored for each frame:

- Angle wheel (i.e., lateral change of the steering wheel)
- Angle pedal (i.e., deflection of the gas pedal)
- Position of the box in pixels
- Size of the box in pixels

After data preparation, different measures can be calculated (e.g., with Matlab):

- Number of lateral and longitudinal error states
- Duration of lateral and longitudinal error states
- Mean lateral position (i.e., tendency to hold the box more to the left or more to the right)
- Mean longitudinal size (i.e., tendency to hold the box smaller or larger compared to the ideal box position)
- Standard deviation of lateral position
- Standard deviation of longitudinal size

Participants’ DRT performance can be determined by calculating the hit rate and the reaction time for correct answers (see [4]).

### Validation studies

Two studies were conducted to verify the validity and sensitivity of the new method regarding its ability to distinguish between various secondary tasks. In a first experiment, the ability of the BT+DRT to assess secondary task demand while driving was compared to the Lane Change Test (LCT) and driving through a simple course in a driving simulator. For visual-manual secondary tasks, the results between the BT and the LCT were roughly comparable. However, compared to the driving simulation task (which was implemented according to the NHTSA test protocol [9]), the BT was much more sensitive to different kinds of secondary tasks. Additionally, it was shown that the DRT is able to cover cognitive distraction effects. For more details, see Morgenstern, Wögerbauer et al. [7].

In a second experiment, the sensitivity of the BT+DRT to different dimensions of driver distraction was examined. The LCT was used as a comparison method. The results showed that the BT can distinguish between visual-manual secondary tasks of different task difficulties. The results were comparable to the results of the LCT. Surprisingly, the BT and the LCT were also sensitive to different levels of cognitive demand, though at a lesser extent than the DRT. For an overview, see Morgenstern, Trommler et al. [8].

### Potential modifications of the BT

Certain parameters of the BT provide opportunities to change the difficulty of the task. For example, modifying the ratio of the boundaries (e.g., a smaller/larger distance between the inner and outer boundary) may lead to an increased/decreased difficulty in maintaining the box within the boundaries. In addition, a modification of the frequency of the sinus functions (longitudinal and lateral cycles per minute) could also be associated with a change in task difficulty. Further research should examine to what extent these changes are related to traffic complexity and whether traffic complexity can be modelled by these parameters. These findings could be useful to apply the new method to different traffic scenarios, such as urban or rural driving.

## Conclusion

In this article, the implementation of the BT with C# and Unity for Microsoft Windows is described. By using the BT in combination with a DRT, visual-manual and cognitive distraction effects caused by secondary task engagement can be assessed differentiated. The BT+DRT is a cost-effective and easy-to-use method, which can be implemented in laboratory settings as well as in driving simulators.

Therefore, the BT+DRT can be used in an early stage of the development process of a new technology (e.g., an in-vehicle infotainment system). Moreover, the BT+DRT could also be used for assessing cognitive or visual-manual impairments due to fatigue, mental illness, or age. Further studies should investigate this. Moreover, in further studies critical thresholds should be defined to be able to judge when a secondary task is too demanding.

Finally, it should be noted that using our own sensors on the steering wheel and gas pedal can raise some issues. In contrast to the use of standard systems, more technical understanding is required for the setup and implementation. Furthermore, the data of standard systems can be more reliable, since the sensors are hard-wired and less exposed to external disruptions (e.g., by touching). However, we decided to use dedicated standard sensors because there are different models of steering wheels and gas pedals that differ in their resolution and retrieval of the data. Arduinos and corresponding sensors are cost-effective and very well documented (i.e., open-source). Thus, our setup provides direct access to raw data, which can be processed in a flexible way depending on the requirements of developers and researchers.

## Appendix Supplementary materials

Image, application 1
